# Concurrent OCT and OCT angiography of retinal neurovascular degeneration in the 5XFAD Alzheimer’s disease mice

**DOI:** 10.1117/1.NPh.8.3.035002

**Published:** 2021-07-10

**Authors:** Tae-Hoon Kim, Taeyoon Son, Dieter Klatt, Xincheng Yao

**Affiliations:** a University of Illinois at Chicago, Department of Bioengineering, Chicago, Illinois, United States; b University of Illinois at Chicago, Department of Ophthalmology and Visual Sciences, Chicago, Illinois, United States

**Keywords:** Alzheimer’s disease, retina, OCT, OCTA, neurovascular degeneration, 5XFAD

## Abstract

**Significance**: As one part of the central nervous system, the retina manifests neurovascular defects in Alzheimer’s disease (AD). Quantitative imaging of retinal neurovascular abnormalities may promise a new method for early diagnosis and treatment assessment of AD. Previous imaging studies of transgenic AD mouse models have been limited to the central part of the retina. Given that the pathological hallmarks of AD frequently appear in different peripheral quadrants, a comprehensive regional investigation is needed for a better understanding of the retinal degeneration associated with AD-like pathology.

**Aim**: We aim to demonstrate concurrent optical coherence tomography (OCT) and OCT angiography (OCTA) of retinal neuronal and vascular abnormalities in the 5XFAD mouse model and to investigate region-specific retinal degeneration.

**Approach**: A custom-built OCT system was used for retinal imaging. Retinal thickness, vessel width, and vessel density were quantitatively measured. The artery and vein (AV) were classified for differential AV analysis, and trilaminar vascular plexuses were segmented for depth-resolved density measurement.

**Results**: It was observed that inner and outer retinal thicknesses were explicitly reduced in the dorsal and temporal quadrants, respectively, in 5XFAD mice. A significant arterial narrowing in 5XFAD mice was also observed. Moreover, overall capillary density consistently showed a decreasing trend in 5XFAD mice, but regional specificity was not identified.

**Conclusions**: Quadrant- and layer-specific neurovascular degeneration was observed in 5XFAD mice. Concurrent OCT and OCTA promise a noninvasive method for quantitative monitoring of AD progression and treatment assessment.

## 1 Introduction

Alzheimer’s disease (AD) is a progressive neurodegenerative disease and the fifth leading cause of death in Americans over 65 years.^1^ The death rate resulting from AD soared 123% between 2000 and 2015,^1^ and it is anticipated that one in every 85 people will be living with AD by 2050.^2^ There is no cure for AD to date; thus, early detection and therapeutic interventions are imperative to prolong cognitive function in AD patients.^3^^,^^4^ A definitive diagnosis of AD can be achieved by cerebrospinal fluid assays or positron emission tomography imaging.^5^^,^^6^ However, these methods are often invasive, costly, time-consuming, and limited in their availability.

There is ample evidence to support that the retina and brain share common pathological hallmarks of AD,^7^^,^^8^ highlighting the potential usefulness of retinal examination for AD diagnosis. Beta-amyloid (Aβ) deposits in the retina were found to be associated with brain Aβ burden,^9^^,^^10^ and morphological and functional impairments of the retina were also observed in AD patients.^11^^,^^12^ Given the clear optics of the eye, the retina offers easy accessibility to ocular imaging modalities, enabling noninvasive, cost-effective, and rapid screening to define at-risk AD populations. Accordingly, over the past decade, substantial progress has been made in retinal imaging techniques, such as retinal fluorescent imaging,^13^ fluorescence lifetime imaging,^14^ hyperspectral imaging,^15^ spectrophotometric fundus imaging,^16^ optical coherence tomography (OCT),^17^ and OCT angiography (OCTA),^18^ to detect AD-associated abnormalities.

With depth-resolved capabilities, OCT and OCTA have been mostly used in clinical studies. OCT illustrated morphological abnormalities in the retina associated with AD,^17^ and OCTA demonstrated functional abnormalities in the retinal vasculature in AD patients.^18^ However, the clinical results often conflicted with each other,^19^^,^^20^ and age-related confounding factors cannot be easily excluded in measurement,^21^^,^^22^ limiting reliable interpretations for retinal changes associated with AD. To better understand the causation of AD in the retina, studies using transgenic mouse models, recapitulating amyloid plaques, neurofibrillary tangles, or neurodegeneration,^23^ have been recently demonstrated. Georgevsky et al.^24^ used spectral-domain OCT in APP/PS1 mice and observed significant inner retinal thinning at 9 months and outer retinal thinning at 12 months. Harper et al.^25^ used multicontrast OCT in APP/PS1 mice and found that retinal vessel density and retinal thickness were comparable between the transgenic mice and control mice at 54 to 103 weeks old. Song et al. conducted an *ex vivo* study using OCT and angle-resolved low-coherence interferometry in 3xTG-AD mice at 15 to 16 months of age. They found significant nerve fiber layer (NFL) thinning and scattering parameters changes on the NFL in 3xTG-AD mice.^26^ Lim et al.^27^ used spectral-domain OCT in 5XFAD mice and found significant NFL thinning at 6, 12, and 17 months of age.

The previous animal studies well demonstrated comparative results between transgenic and control mice; however, their quantitative analysis was mainly performed at the central retina, i.e., the optic nerve head (ONH) region. Since pathological hallmarks frequently appear in different peripheral quadrants of the human retina in AD,^13^^,^^20^^,^^21^^,^^28^^,^^29^ comprehensive regional monitoring would provide better insights into AD-associated retinal degeneration. Moreover, comparative imaging of retinal neuronal and vascular systems may become a useful tool to study pathological mechanisms of AD-associated retinal degeneration in different mouse models. In this study, concurrent OCT/OCTA monitoring of retinal neuronal and vascular abnormalities in all retinal quadrants (up to ∼30-deg eccentricity from the ONH) in the 5XFAD mouse model of AD was first-time demonstrated. A clear trend toward a reduction in both retinal thickness and vascular parameters was found in different regions of the 5XFAD mouse retina.

## 2 Materials and Methods

### 2.1 Animals

Six-month-old female 5XFAD mice (N=5) and B6SJLF1/J mice (WT; N=6) were used in this study. 5XFAD mice overexpress mutated versions of the human amyloid precursor protein (APP) and human presenilin-1, resulting in Aβ plaques in the brain and retina.^30^ It is well documented that 6-month-old 5XFAD mice showed behavioral deficits,^31^ neuronal death,^32^ capillary stalling,^33^ and abnormal tissue viscoelasticity in brain subregions.^34^ All the mice were directly obtained from the Mutant Mouse Resource and Research Center (MMRRC Stock No. 34840-JAX; Jackson Laboratory, Bar Harbor, Maine) after genotyping for the ${\mathrm{Pde}6b}^{\mathrm{rd}1}$ mutation. Thus, the 5XFAD mice homozygous for the recessive ${\mathrm{Pde}6b}^{\mathrm{rd}1}$ allele were not included in this study to ensure that visual impairments did not affect results.

### 2.2 Imaging System

A custom-designed spectral-domain OCT system was used in this study. The system has been employed in multiple functional OCT/OCTA studies.^35^^–^^37^ Briefly, a near-infrared superluminescent diode (λ=810  nm; Δλ=100  nm; D-810-HP, Superlum, Carrigtwohill, County Cork, Ireland) was used as a light source. A line CCD camera with 2048 pixels (AViiVA EM4; e2v Technologies, Chelmsford, United Kingdom) was used for recording OCT spectra in the custom-built OCT spectrometer. The frame rate of the camera was set to 50 kHz. The axial and lateral resolutions were theoretically estimated as 2.9 and 11  μm, respectively. The measured axial resolution from the scattering profile of the external limiting membrane (ELM) was 3.4  μm. The ELM is the thin line of junctional complexes between Müller cells and photoreceptors (<1  μm thickness), which can be well described by a Gaussian to confirm the axial point spread function.^38^ 1-mW power was illuminated on the mouse cornea.

### 2.3 Experimental Procedures

Anesthesia was intraperitoneally induced by a mixture of ketamine (100  mg/kg body weight) and xylazine (5  mg/kg body weight), and a drop of 1% tropicamide ophthalmic solution (Akorn, Lake Forest, Illinois) was applied to the imaging eye. Next, a cover glass (12-545-80; Microscope cover glass, Fisherbrand, Waltham, Massachusetts) with a drop of eye gel (Severe; GenTeal, Novartis, Basel, Switzerland) was placed on the imaging eye. After the mouse was fully anesthetized, the head was fixed by a bite bar and ear bar in the animal holder that provided five degrees of freedom (i.e., x, y, z, pitch, and roll). Volumetric raster scans were individually acquired from each retinal quadrant (dorsal, nasal, temporal, ventral quadrant, and the ONH). The ONH region was first captured, which can serve as a central point of the retina, and the dorsal/ventral quadrants were captured, followed by the nasal/temporal quadrants. Four repeated B-scans at each slow-scan position were collected for OCTA construction; thus, each OCT volume consisted of 4×600×600 A-scans. All animal experiments were approved by the local animal care and biosafety office and performed following the protocols approved by the Animal Care Committee (ACC) at the University of Illinois at Chicago (ACC Number: 19-044). This study followed the Association for Research in Vision and Ophthalmology Statement for the Use of Animals in Ophthalmic and Vision Research.

### 2.4 OCTA Image Processing

**Fig. 1:**
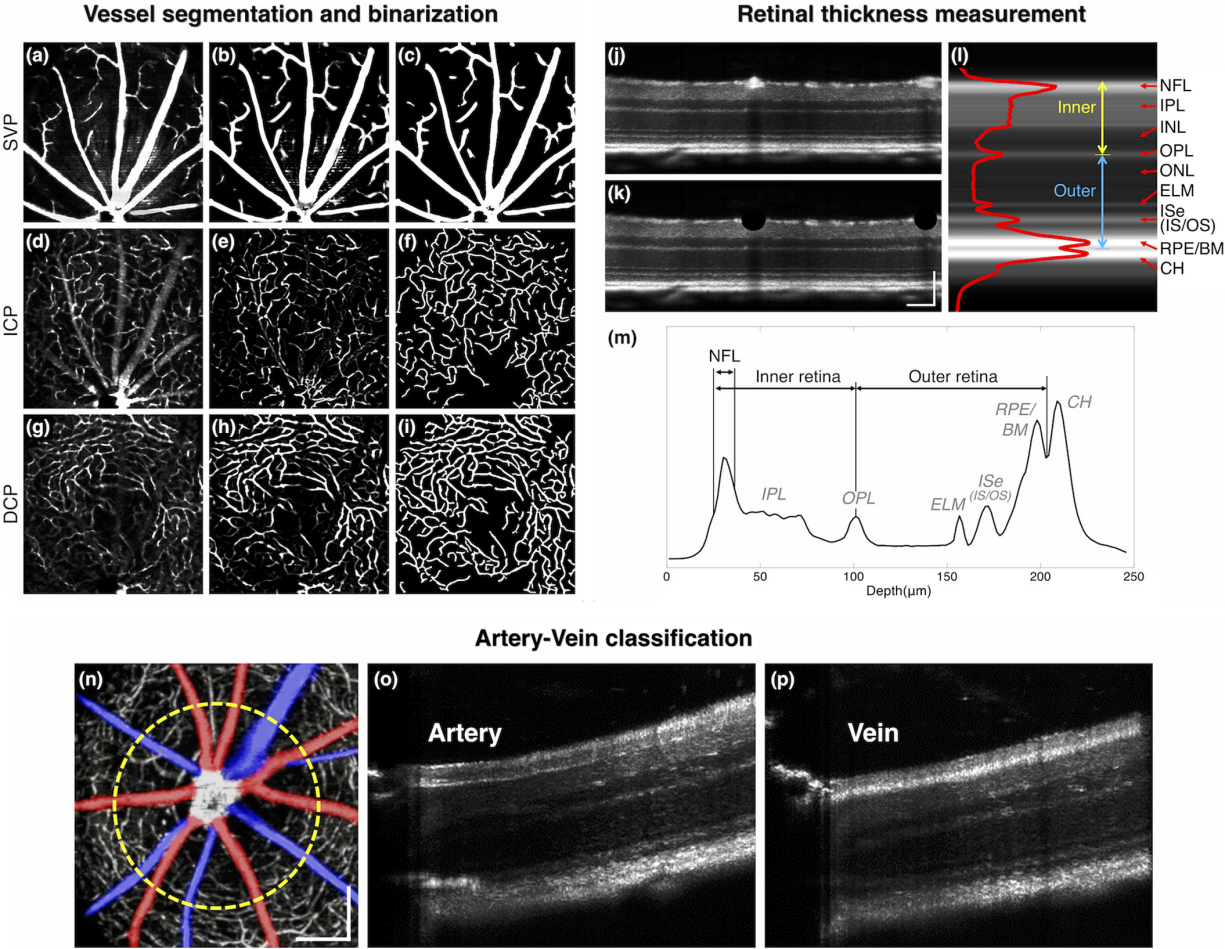
OCTA binarization procedures. Representative *en face* OCTA of (a) SVP, (d) ICP, and (g) DCP in dorsal quadrant of the mouse retina. (b), (e), (h) Vessel enhancement was performed, followed by (c), (f), (i) binarization processing. (j) Flattened OCT B-scan. (k) Flattened OCT B-scan after vessel removal. Scale bars: 100  μm. (l) Representative A-line intensity profile. (m) Thickness measurement points. Midpoints of ascending and descending slopes of the NFL intensity profile, the OPL peak point, and the RPE/CH trough point were used in the measurement. (n) Pseudocolored *en face* OCTA image (red for arteries and blue for veins). Vessel width was measured at FWHM of the intensity profile of the yellow dashed circle. Scale bars: 200  μm. Representative pseudoradial OCT B-scans illustrate the different reflectance profiles between the (o) artery and (p) vein. NFL, nerve fiber layer; IPL, inner plexiform layer; INL, inner nuclear layer; OPL, outer plexiform layer; ONL, outer nuclear layer; ELM, external limiting membrane; ISe, inner segment ellipsoid; IS, inner segment; OS, outer segment; RPE, retinal pigment epithelium; BM, Bruch’s membrane; CH, choroid.

The OCTA images were constructed by implementing intensity-based speckle variance processing.^39^ Next, retinal flattening was performed by realigning each A-line. Three vascular layers were then manually segmented for vessel density analysis (Fig. 1), i.e., the superficial vascular plexus (SVP), intermediate capillary plexus (ICP), and deep capillary plexus (DCP).^40^ For each segmented layer, en face OCTA image was reconstructed by maximum intensity projection, and a Hessian-based vessel enhancement method proposed by Jerman was used to enhance vasculature in each en face image.^41^ After that, binarization for the SVP was done by adaptive thresholding, and binarization for the ICP and DCP was done by ridge-based vessel detection method.^42^ All binarized images were further processed by morphological opening operation to remove small particle noises. Image processing was performed on MATLAB R2016a (MathWorks, Natick, Massachusetts), in coordination with image processing package available in Fiji software.^43^

### 2.5 Data and Statistical Analysis

Retinal thickness, vascular width, and vascular density were measured for quantitative comparison. For thickness measurement, flattened OCT-B scans were prepared, and large vessels were manually removed to measure the retinal thickness solely [Fig. 1(k)]. Central 200 A-lines were averaged in the horizontal direction, returning one averaged intensity profile [Fig. 1(l)]. The regions in where the vessels were manually removed [Fig. 1(k)] were excluded from the averaging. Based on the average profile, the NFL, inner retinal layer, outer retinal layer, and total retinal thickness were manually measured [Fig. 1(m)]. For vascular width measurement, the artery and vein (AV) around the ONH were first classified by referring vascular morphology on radially resliced OCT B-scans,^44^ and the vessel width of all first branches was measured based on the circular profile, 300  μm away from the ONH [Figs. 1(n)–1(p)]. The full-width at half-maximum (FWHM) was measured using the “findpeaks” function (MATLAB R2016a, MathWorks, Natick, Massachusetts) in determining the vessel diameter. The vessel density for each plexus was defined as the percentage of area occupied by vasculature in the binarized OCTA images. The two-sample t-test was performed for the statistical comparison between WT and 5XFAD, and a p-value<0.05 was considered statistically significant. In this study, each quadrant measurement was treated as an independent sample based on the current understanding that AD-associated retinal degeneration appears in a specific retinal region, and this local effect does not affect measurements for other quadrant observations.^20^^,^^21^ Statistical analysis was performed on Origin 2020b (OriginLab, Northampton, Massachusetts).

## 3 Results

**Fig. 2:**
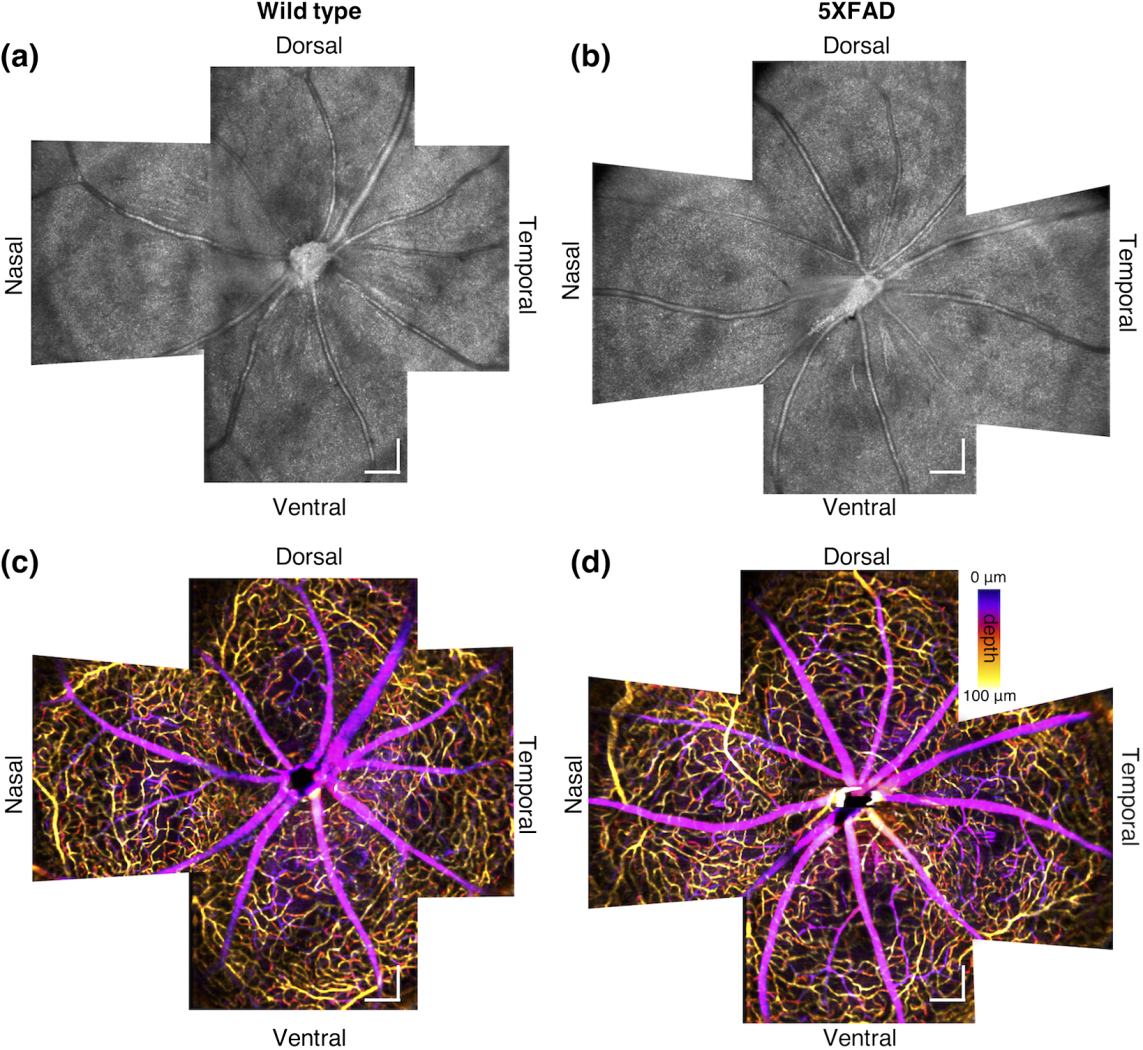
Wide-field OCT and OCTA montages of (a), (c) the WT and (b), (d) the 5XFAD mouse retina. The color bar indicates the depth scale from the retinal surface to the outer plexiform layer. Scale bars: 200  μm.

Figure 2 shows wide-field OCT and OCTA montages, consisting of four retinal quadrant images of a WT and 5XFAD mouse. Total imaging area covers ∼2.2  mm×2.2  mm of the central retina, which is ∼61-deg visual angle by the conversion factor of 36  μm per degree of visual angle. The conversion factor was estimated based on a recent work demonstrating the adult mouse eye model incorporated with a contact lens and gel thickness information.^45^ This field of view can partially visualize network topologies of the mouse retina vasculature. Arteries and veins radially emanate from the ONH, whereas the capillary plexus reveals a circular mesh structure centered at the ONH.^46^ Retinal abnormalities were not recognized between the WT and 5XFAD mouse through visual inspection, implying only subtle changes might occur in the 5XFAD mouse retina. Each quadrant OCT and OCTA volume was then individually processed and analyzed for the quantitative assessment.

**Fig. 3:**
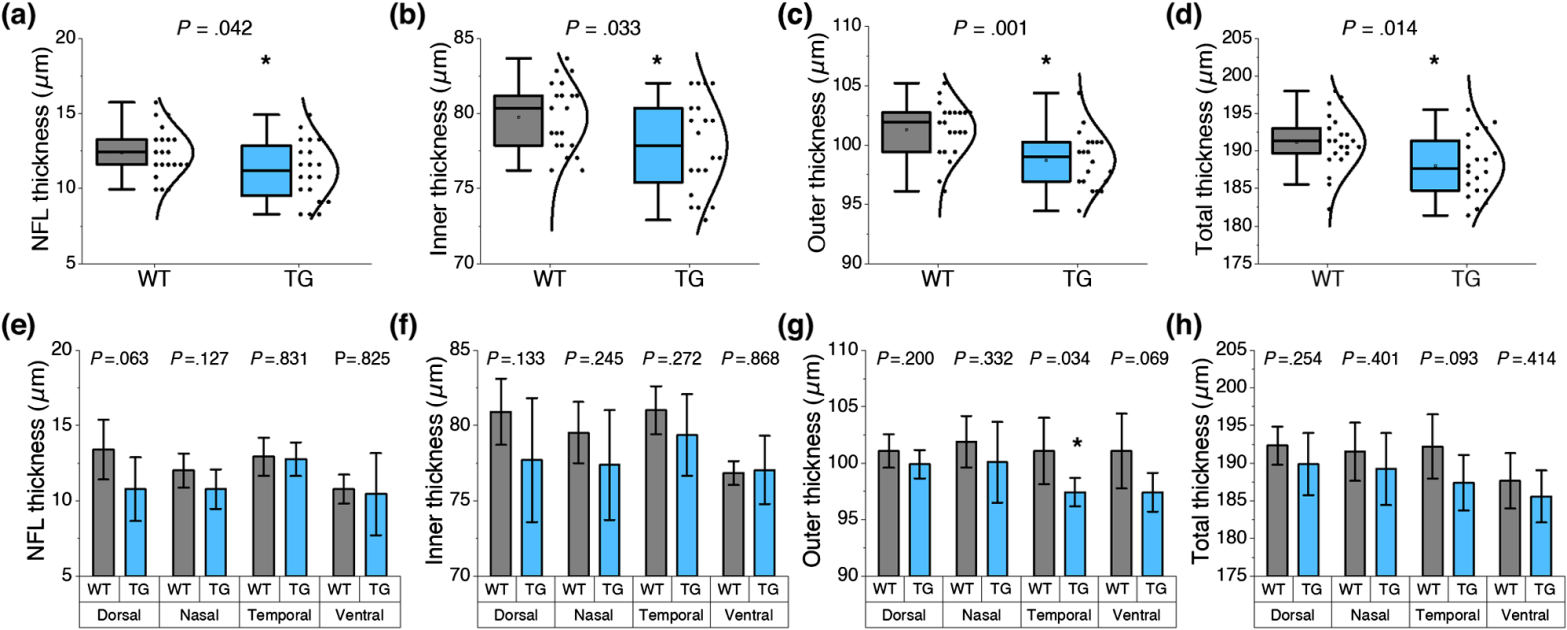
Comparative retinal thickness measurement. (a) Overall NFL thickness and (e) quadrant specific measurement. (b) Overall inner retinal thickness and (f) quadrant-specific measurement. (c) Overall outer retinal thickness and (g) quadrant-specific measurement. (d) Overall total retinal thickness and (h) quadrant-specific measurement. Black dots in (a)–(d) indicate individual data points measured from each retinal quadrant. Data are expressed as mean±standard deviation per group. A two-sample t-test was performed for intergroup comparison. P-values are indicated in each plot, and statistical significance is indicated by an asterisk: *P<0.05. N=6 for WT (wild-type) and N=5 for TG (transgenic 5XFAD).

We first analyzed neuronal change by retinal thickness measurement. The thickness of the NFL, inner retina, and outer retina were separately measured at the consistent position, ∼600  μm away from the ONH in each quadrant. Overall, the NFL thickness (WT: 12.4±1.6  μm; 5XFAD: 11.2±2.0  μm; p=0.042), inner retinal thickness (WT: 79.8±2.3  μm; 5XFAD: 77.9±3.1  μm; p=0.033), and outer retinal thickness (WT: 101.3±2.3  μm; 5XFAD: 98.7±2.4  μm; p=0.001) were significantly reduced in 5XFAD mice (Fig. 3). Accordingly, total retinal thickness was significantly lower in 5XFAD mice compared with WT mice (WT: 191.2±3.8  μm; 5XFAD: 188.0±4.1  μm; p=0.014). Quadrant analysis also showed a consistent retinal thinning in different regions. There seems to be a trend that the dorsal area in the inner retina showed an advanced retinal thinning compared with the ventral side. The NFL in the dorsal quadrant was notably thinner in 5XFAD mice (WT: 13.4±2.0  μm; 5XFAD: 10.8±2.1  μm; p=0.063) [Fig. 3(e)]. Another notable observation is the reduction of the outer retinal thickness. All quadrants showed a reduction in the outer retina, and the difference in the temporal quadrant was statistically significant (WT: 101.1±2.9  μm; 5XFAD: 97.4±1.3  μm; p=0.034) [Fig. 3(g)]. Collectively, retinal thinning in all quadrants was clearly demonstrated in 6-month-old 5XFAD mice, and retinal thinning was attributed not only to the inner retina but also to the outer retina.

**Fig. 4:**
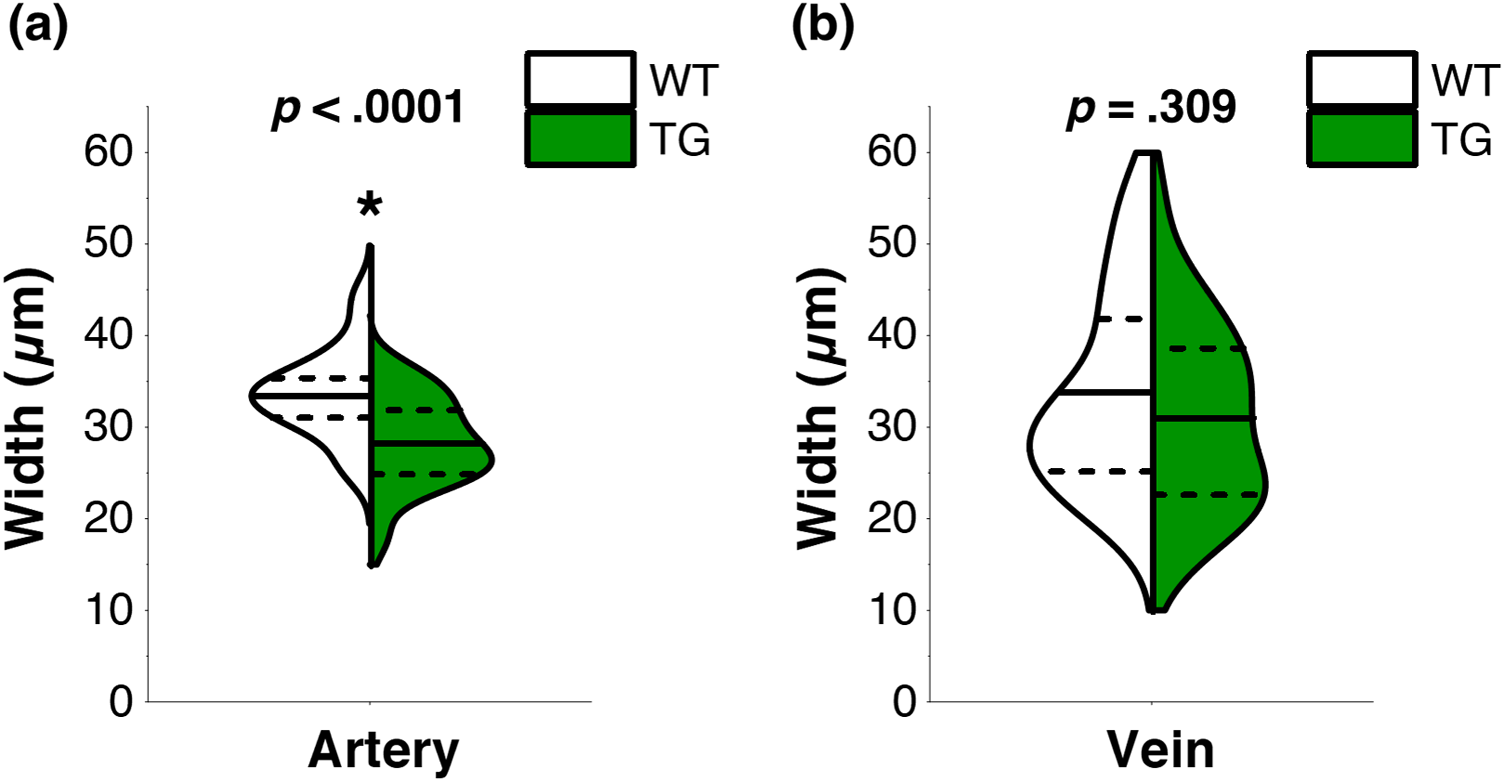
Split-violin plots of (a) arterial width measurement and (e) venular width measurement. Data are expressed as mean±standard deviation per group. A two-sample t-test was performed for intergroup comparison. P-value for statistical significance is indicated by an asterisk. *P<0.05. N=6 for WT (wild-type) and N=5 for TG (transgenic 5XFAD).

We next investigated vascular changes. To quantify the vascular narrowing/widening effect, we measured arterial and venular width following the circular profile around the ONH with radii of 300  μm. AV classification was achieved by the pseudoradial scanning method.^44^ Figures 1(o) and 1(p) show the radial OCT B-scans showing hyperreflective wall boundaries in retinal arteries, whereas these wall boundaries were absent in retinal veins. We found a significant arterial narrowing in 5XFAD mice (WT: 33.6±4.7  μm; 5XFAD: 28.3±4.8  μm; p<0.0001 for average arterial width comparison, p=0.007 for average arterial width per mouse comparison, i.e., the average of the mean of each mouse dataset) [Fig. 4(a)], and the venular width was comparable between WT and 5XFAD mice (WT: 33.9±10.6  μm; 5XFAD: 31.1±10.1  μm; p=0.309 for average venular width comparison, p=0.201 for average venular width per mouse comparison, i.e., the average of the mean of each mouse dataset) [Fig. 4(b)]. The venular width exhibited more variations than the arterial width and did not show significant alterations between the two strains.

**Fig. 5:**
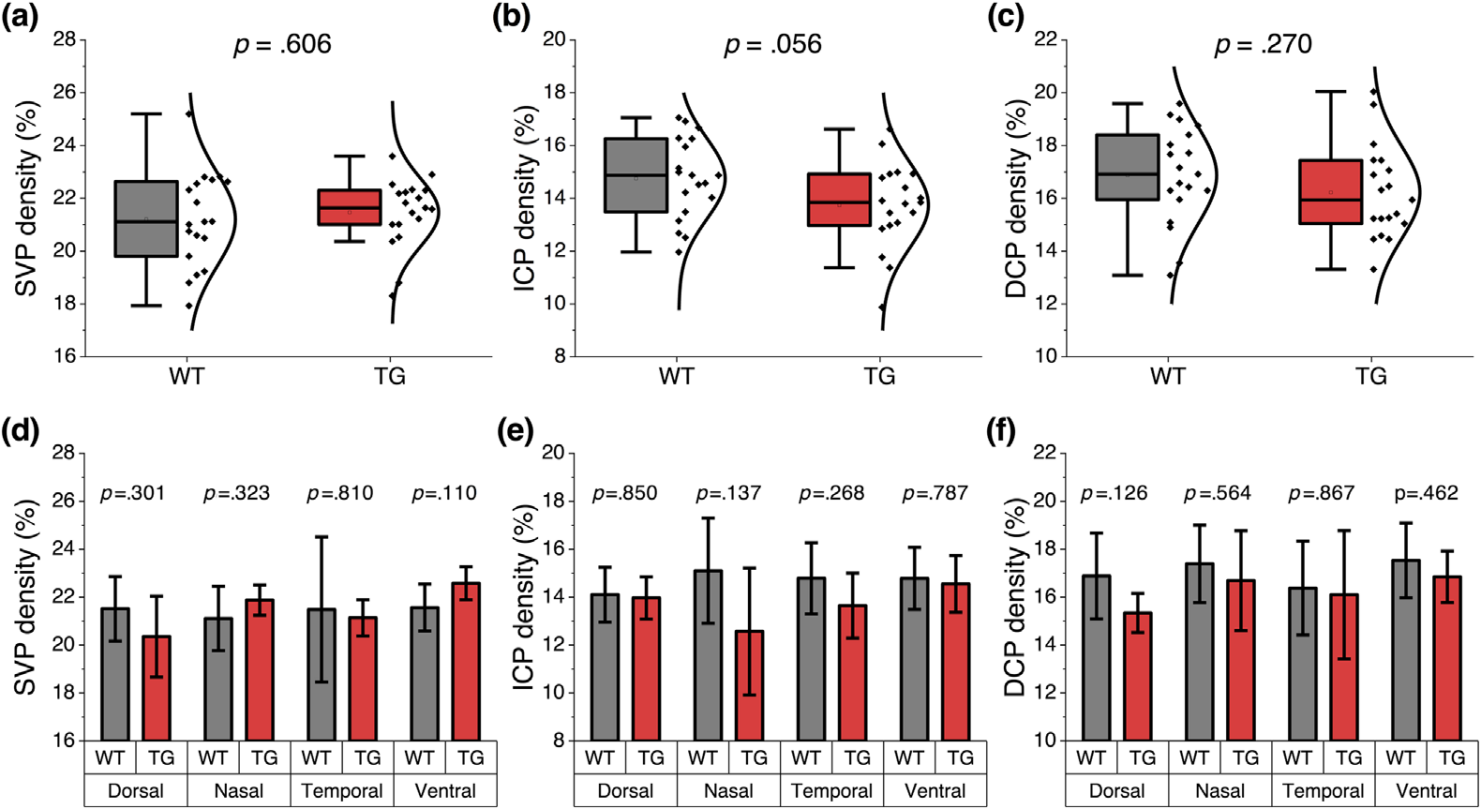
Comparative vessel density measurement. (a) Overall SVP density and (d) quadrant-specific measurement. (b) Overall ICP density and (e) quadrant-specific measurement. (c) Overall DCP density and (f) quadrant-specific measurement. Black dots in (a)–(c) indicate individual data points measured from each retinal quadrant. Data are expressed as mean±standard deviation per group. A two-sample t-test was performed for intergroup comparison. P-values are indicated in each plot. N=6 for WT (wild-type) and N=5 for TG (transgenic 5XFAD).

Next vascular density was analyzed. The vasculature observed in OCTA reflects functional vessels.^47^ To measure the layer-specific change, trilaminar vascular plexuses were segmented (Fig. 1). Figure 5 shows that the vessel density in the SVP was comparable between WT and 5XFAD mice (WT: 21.2±1.8%; 5XFAD: 21.4±1.3%; p=0.606), whereas the overall capillary density showed a decreasing trend in 5XFAD mice. Especially, the ICP density reduction in 5XFAD mice was close to being statistically significant (WT: 14.7±1.5%; 5XFAD: 13.7±1.6%; p=0.056) [Fig. 5(b)]. We also found that capillary density consistently showed a decreasing trend in all retinal quadrants, although quadrant specificity tends to vary [Figs. 5(e) and 5(f)]. Collectively, the results demonstrate that the arterial narrowing effect was significant in 5XFAD mice, and functional capillary density in all quadrants was reduced in 5XFAD mice.

## 4 Discussion

In this study, region-specific neurovascular degeneration was found in 6-month-old 5XFAD mice. Significant inner retinal thinning was first noted in 5XFAD mice (Fig. 3), consistent with a recent finding of selective inner retinal deficits in 5XFAD mice at 6 months.^27^ The retinal thinning generally indicates neuronal death,^48^ and increasing evidence suggests that Aβ deposits in the inner retina may be associated with degeneration of retinal ganglion cells.^49^ Especially, the 5XFAD mouse model was found with the highest concentration of Aβ peptides in the brain and retina among AD mouse models.^50^ Although the exact role of Aβ in AD pathology remains an open question, it is believed that aggregation of extracellular Aβ can disrupt cellular communication and activities, which can damage synapses and ultimately induce neuronal cell death.^51^ In a recent study, notable Aβ accumulation was found around degenerating retinal ganglion cells in AD subjects.^9^ Also, we confirmed consistent outer retinal thinning in all quadrants [Fig. 3(g)]. The reason can be attributed to aggressive Aβ deposition not only in the inner retina but also in the outer retina in 5XFAD mice. It was reported that Aβ was largely accumulated in the 5XFAD retina under the retinal pigment epithelium (RPE), which might cause inflammation and contribute to photoreceptor cell death.^52^^,^^53^ Likewise, increased Aβ deposits around the rod photoreceptors were recently observed in AD subjects along with outer retinal thinning.^13^^,^^54^ The presence of Aβ may cause defects in photoreceptor cell function and potentially contribute to visual impairments in AD subjects as reflected by the reduction of contrast sensitivity, visual acuity, color vision, and visual field.^55^

Six-month-old 5XFAD mice also manifested vascular abnormalities in the retina. Arteries showed a significant narrowing in 5XFAD mice [Fig. 4(a)], which might be a consequence of the capillary flow deficits. Although capillary density reduction did not reach statistical significance, the ICP and DCP consistently exhibited a decreasing trend in all quadrants of the 5XFAD mouse retina [Figs. 5(b) and 5(c)]. Hernández et al.^33^ found that capillary stalling was elevated in 6-month-old 5XFAD mice and described that a small portion of capillary occlusion could dramatically decrease blood flow in up-and-down-stream vessels, resulting in the blood flow changes on cerebral blood flow. Nortley et al. also suggested that capillaries could be the most critical locus where Aβ can decrease cerebral blood flow,^56^ and reduced blood flow was found to increase in Aβ deposition, suggesting that blood flow deficits can further worsen Aβ pathology.^57^ However, there is an opposite pathophysiological basis that angiogenesis can occur in response to impaired cerebral perfusion and vascular injury.^58^ Such inflammation due to Aβ accumulation can induce the retina to become hypoxic and trigger angiogenesis,^19^ which can cause increased vessel density observed in OCTA.^59^ A recent clinical study reported capillary density increase in AD subjects,^60^ while others reported the opposite results.^61^^,^^62^ We speculate that capillary density may increase at the initial stage of the Aβ accumulation. However, at the later stage, when neuronal cell loss occurs, capillary density may naturally decrease due to a reduction of metabolic demand and damaged endothelial cells. A further longitudinal study is required to verify this hypothesis.

Individual quadrant OCT/OCTA analysis suggested that neurovascular degeneration may occur in all retinal regions, but there seems to be a trend of regional specificity in terms of degeneration progress. Retinal thinning of the NFL and outer retina was distinguished in the dorsal and temporal quadrant, respectively, in 5XFAD mice (Figs. 3 and 5). Song et al.^26^ also found different light scattering parameters only in the dorsal quadrant in AD mouse retinas. In fact, a region-specific degeneration is not only limited to the animal models but also appeared in human subjects with AD.^21^ Previous studies demonstrated that retinal changes were predominantly focused on the superior and inferior regions of the NFL in AD subjects.^28^^,^^29^ Querques et al.^12^ reported that ganglion cell layer thickness was reduced in AD subjects only in the central and temporal retinal region. Koronyo et al.^13^ found that Aβ plaques mainly appeared in the periphery of the superior quadrant, whereas some regional changes were discovered in each retinal layer differentially.^54^ Such discrepancy requires further verification of region-specific changes in the retina associated with AD pathology.

This study has three limitations to be addressed. First, a relatively small sample size was used in this cross-sectional study, which naturally lowered the statistical power to draw a definitive conclusion. Prospective longitudinal monitoring with an increasing sample size would further elucidate retinal abnormalities associated with AD pathology in transgenic mouse models. Second, there was no conclusive biomarker confirmation. Postmortem histopathology would be helpful to confirm the presence of AD biomarkers, such as Aβ42, total tau (T-tau), and phosphorylated tau (P-tau), in different retinal regions to correlate the level of biomarkers with OCT/OCTA features. Third, it is important to be aware of anesthesia-related cardiovascular effects, including changes in blood pressures, cardiac output, and varying heart rhythms. The ketamine–xylazine mixture is known to have potent cardiodepressive effects that can potentially lower local vessel diameter and blood flow over time.^63^^,^^64^ Since the effect is time-dependent, in this study, the ONH region was always first captured in each mouse, and vessel width measurement was performed around the ONH [Fig. 1(n)]. Monitoring vital signs during image acquisition would allow for better understanding of anesthesia-related cardiovascular effects on the mouse retina. Despite the limitations of this study, *in vivo* full quadrant imaging may provide a foundation for future detailed exploration of the relationship between retinal neurovascular defects and AD-associated retinal degeneration.

## 5 Conclusions

This study demonstrates *in vivo* OCT/OCTA monitoring of all retinal quadrants, which allowed examining region-specific neurovascular degeneration in the 5XFAD mouse retina. Six-month-old 5XFAD mice revealed that inner and outer retinal degeneration were explicitly advanced in the dorsal and temporal quadrant, respectively. The arterial narrowing was observed in 5XFAD compared with wild-type mice. Overall decrease of measured capillary density was also observed in 5XFAD mice, suggesting a tight correlation between retinal neurons and vasculature. The proposed imaging strategy promises a noninvasive method for longitudinal monitoring of AD progression and treatment assessment in transgenic mouse models of AD.

## Code, Data, and Materials Availability

The data that support the findings of this study are available from the corresponding author upon reasonable request.
